# Behavioral Characteristics of Ubiquitin-Specific Peptidase 46-Deficient Mice

**DOI:** 10.1371/journal.pone.0058566

**Published:** 2013-03-05

**Authors:** Saki Imai, Makoto Kano, Keiko Nonoyama, Shizufumi Ebihara

**Affiliations:** Division of Biomodeling, Graduate School of Bioagricultural Sciences, Nagoya University, Nagoya, Japan; Hokkaido University, Japan

## Abstract

We have previously identified *Usp46,* which encodes for ubiquitin-specific peptidase 46, as a quantitative trait gene affecting the immobility time of mice in the tail suspension test (TST) and forced swimming test. The mutation that we identified was a 3-bp deletion coding for lysine (Lys 92), and mice with this mutation (MT mice), as well as *Usp46* KO mice exhibited shorter TST immobility times. Behavioral pharmacology suggests that the gamma aminobutyric acid A (GABA_A_) receptor is involved in regulating TST immobility time. In order to understand how far *Usp46* controls behavioral phenotypes, which could be related to mental disorders in humans, we subjected *Usp46* MT and KO mice to multiple behavioral tests, including the open field test, ethanol preference test, ethanol-induced loss of righting reflex test, sucrose preference test, novelty-suppressed feeding test, marble burying test, and novel object recognition test. Although behavioral phenotypes of the *Usp46* MT and KO mice were not always identical, deficiency of *Usp46* significantly affected performance in all these tests. In the open field test, activity levels were lower in *Usp46* KO mice than wild type (WT) or MT mice. Both MT and KO mice showed lower ethanol preference and shorter recovery times after ethanol administration. Compared to WT mice, *Usp46* MT and KO mice exhibited decreased sucrose preference, took longer latency periods to bite pellets, and buried more marbles in the sucrose preference test, novelty-suppressed feeding test, and marble burying test, respectively. In the novel object recognition test, neither MT nor KO mice showed an increase in exploration of a new object 24 hours after training. These findings indicate that *Usp46* regulates a wide range of behavioral phenotypes that might be related to human mental disorders and provides insight into the function of USP46 deubiquitinating enzyme in the neural system.

## Introduction

The CS mouse, an inbred strain originally established by crossing the NBC and SII strains (both now extinct), exhibits extremely low immobility time in both the tail suspension test (TST) and forced swimming test (FST). Previously, we performed quantitative trait locus (QTL) genetic analysis using an F2 population cross between CS and normal C57BL/6J mice to identify the gene responsible for the reduced immobility times. After mapping the QTL, we produced several congenic or subcongenic strains, in order to narrow the QTL interval, and focused on a candidate gene within this QTL region. We produced bacterial artificial chromosome transgenic mice, and finally rescued the phenotype. Consequently, we identified *Usp46,* which encodes an ubiquitin-specific peptidase, as one of the genes responsible for the shortened immobility times [1].

The *Usp46* mutation that we identified has a 3-bp deletion coding for lysine (Lys 92) in the open reading frame, and mice with this mutation (MT mice) exhibit a shorter duration of loss of righting reflex caused by muscimol (gamma aminobutyric acid A [GABA_A_] receptor agonist) administration and a significantly lower amplitude of the muscimol-induced GABA_A_ current in hippocampal CA1 pyramidal neurons. Additionally, hippocampal expression of the 67-kDa isoform of glutamic acid decarboxylase is decreased in these mice. Thus, this mutation is implicated to play a role in the regulation of the GABAergic system. To support these results, we examined null *Usp46* (*Usp46* KO) mice, and found that TST immobile behavior of the *Usp46* KO mice is similar to the 3-bp deleted *Usp46* mutation and that the GABA_A_ receptor participates in the regulation of immobility times [2]. Although USP46 deubiqutinating enzyme seems to be implicated in the GABAergic system, most of the cellular functions of this enzyme are not known. To understand these issues, the identification of the target substrates of USP46 is critical, but it has not yet been determined. However, recently it is reported that many proteins interact with USP46, which seems to be necessary to regulate biological functions by this enzyme [3]–[5].

In the present study, in order to gain insight into the function of USP46 in the central nervous system, we subjected *Usp46* MT and KO mice to a variety of behavioral tests, particularly taking notice of GABA-related behaviors.

## Materials and Methods

### Animals

We purchased C57BL/6J (WT) mice from CLEA Japan Inc. Animals (all male mice, aged 8–12 weeks) were housed under a 12 hour light/dark cycle (LD 12∶12, 7:00 on, 19:00 off), with free access to food and water in our animal facility, at a temperature maintained at approximately 24°C. Animal care and all experimental procedures were approved by the Animal Experiment Committee, Graduate School of Bioagricultural Sciences, Nagoya University (approved no. 2011030303).

### *Usp46* mutant mice

The *Usp46* mutant mice were developed as congenic strains on a C57BL/6J (B6) genetic background using a marker-assisted breeding strategy. These mice (B6.CS-Ngu1053) contained chromosome 5 regions harboring the *Usp46* of the CS mice [1].

### *Usp46* KO mice

The *Usp46* KO mice were generated as previously described [2]. The KO mice (17-13906 *Usp46* gene trapped mice, TG Resource Bank #6072) were descendants of the mouse strain generated by Trans Genic Inc. (Kumamoto, Japan) using the gene trap technique. Tested mice were the offspring of heterozygous pairings and were backcrossed to B6 a minimum of 9 times in order to remove any phenotypic variations caused by a different genomic background. To determine their genotypes, ear biopsies were performed at 4 weeks of age, and the samples were subjected to DNA detection using PCR. F1 heterozygous and B6 alleles were amplified using a neo primer pair: 5′-CTGAATGAACTGCAGGACGAG-3′ and 5′-GTCCAGATCATCCTGATCGAC-3′, and lox-SA primer pair: 5′-AGGTCGAGGGACCTATACCG-3′ and 5′-GAGGCCGCTTGTCCTCTTTG-3′. The F1 heterozygous mice were crossed between themselves to obtain the F2 generation: wild-type, heterozygous, and homozygous. To determine their genotypes, FP3 primer 5′-ATAGACTCTGCTGTTTCTCCTATGCTCC-3′, RP1 primer 5′-AATGTTGAGGCAAAGCTGCCAAGCTCAC-3′, and SA6AS primer 5′-CCGGCTAAAACTTGAGACCT-3′ were used.

### Behavioral testing

Prior to behavioral testing, mice were moved to an animal room adjacent to the testing room, where their behaviors were assayed. The mice were kept there for 1 week under the same conditions as those in the animal facility. We assayed mice during the light phase (11:00–16:00), after at least 2 hours of adaptation to the test room.

### Open field test

Each mouse was placed in the center of a gray plastic box (40×40×40 cm) and was allowed to freely explore for 5 min, under 40- to 50-lux fluorescent light. During the test, the time spent in the outer (7.5 cm in width) and inner zones (the rest of the outer zone) of the open field was measured using SMART software (version 2.0, Panlab, Spain). Because mice often rested in the outer zone, the nonmoving time was also measured. Additionally, the number of grooming, rearing, and climbing (standing on hind legs with forefeet on the wall) events were scored (because only a few instances of rearing were observed, rearing and climbing were combined). After the open field test, the total distance of movement in the box was calculated. At the end of the test, the mouse was returned to its home cage, and the apparatus was cleaned with 70% ethanol.

### Elevated plus-maze test

The elevated plus-maze consists of 2 open arms (width, 8 cm; length, 25 cm) and 2 closed arms (width, 8 cm; height, 20 cm; length, 25 cm) that extend from a common central platform (8×8 cm) [6]–[7]. This apparatus was elevated to a height of 50 cm above the floor. Each mouse was placed on the center square, facing an open arm, and allowed to freely explore for 6 min under a 40- to 50-lux fluorescent light. The total time spent in the open arm was scored.

### Ethanol preference test

Ethanol preference was measured using the standard two-bottle choice experiment [8]. Individually housed mice were presented with 2 bottles containing water for 5 days. No significant differences in total water consumption were observed between WT, MT, and KO mice (data not shown). After water consumption for 5 days, mice were presented with 2 bottles: one with water and one with 10% (v/v) ethanol solution for 5 days. The positions of the bottles were switched every 24 hours to control for any side preference. The ethanol preference ratio was calculated as the volume of ethanol consumed/total volume of fluid consumed per day.

### Ethanol-induced loss of righting reflex test

Each of the mice was injected with a dose of 3.5 g/kg ethanol (20% v/v in saline intraperitoneally) and was tested for the ethanol-induced loss of righting reflex by placing the mouse on their back in a V-shaped trough [9]–[10]. The loss and recovery of the righting reflex were defined as the inability and ability of the mouse to right itself 3 times within 30 s, respectively.

### Sucrose preference test

Sucrose preference was assessed using the same method as that used for ethanol preference, a two-bottle choice experiment [11]. Mice were presented with 2 bottles: one containing water and the other containing 10% (v/v) sucrose for 5 days. Sucrose preference ratio was calculated using an equation similar to the one used for the ethanol preference test. Because the latency period for drinking sucrose after presentation of the sucrose bottle was different between the genotypes, we measured this parameter on the final day of the sucrose preference test. In this test, when drinking behavior was not observed within 5 min, the latency period was defined as 5 min.

### Novelty-suppressed feeding test

Individually housed mice were weighed, and food was removed from the cage before the test (water remained available *ad libitum*). Twenty-four hours after the food was removed, the mice were habituated to the test room for 2 hours before testing. The mouse was placed in a gray plastic box (40×40×40 cm) containing clean wood chip bedding. A white circular filter paper (90 mm in diameter) was placed in the center of the plastic box, and 2 food pellets were placed on the paper [12]. The latency time to biting the pellets, feeding time, and food consumption were recorded (the maximum latency time was 10 min). Immediately after the test, the mice were removed from the plastic box and transferred to their home cage with a pre-weighed piece of food pellet, where food consumption and feeding time were measured for 5 min under *ad libitum* conditions.

### Marble burying test

Mice were placed individually in the center of a gray plastic box (40×40×40 cm) containing 2-cm deep clean wood chip bedding, with 25 glass marbles (1.7 cm in diameter, yellow) that were evenly distributed. Mice were observed during a 30-min test period. After the test, the number of buried marbles (defined as at least two-thirds of the surface being covered with sawdust) was recorded [13].

### Novel object recognition test

On the first and second days, mice were placed in a gray plastic box (40×40×40 cm) and were allowed to explore freely for 10 min. On the third day (training), 2 identical objects (gray plastic cubes, 5×5×5 cm) were introduced into the box. The mice were again allowed to explore freely for 10 min. After training, the objects and the box were cleaned using 70% ethanol. On the final day (test), one of the cubes was replaced with a novel object (a gray plastic quadrangular pyramid, each side of the base was 5 cm). Mice were placed in the box again, and explored for 10 min. The number of instances of contact with each object and the total time spent exploring each object were recorded. To avoid any preference for place in the box, the position of each object was changed between each trial [14]–[15].

### Statistics

All data are expressed as the mean+S.E.M. Statistical significance was determined by one-way ANOVA with the Fisher's PLSD test for multiple comparisons and student's *t*-tests for 2-group comparisons.

## Results

### Open field test

**Figure 1:**
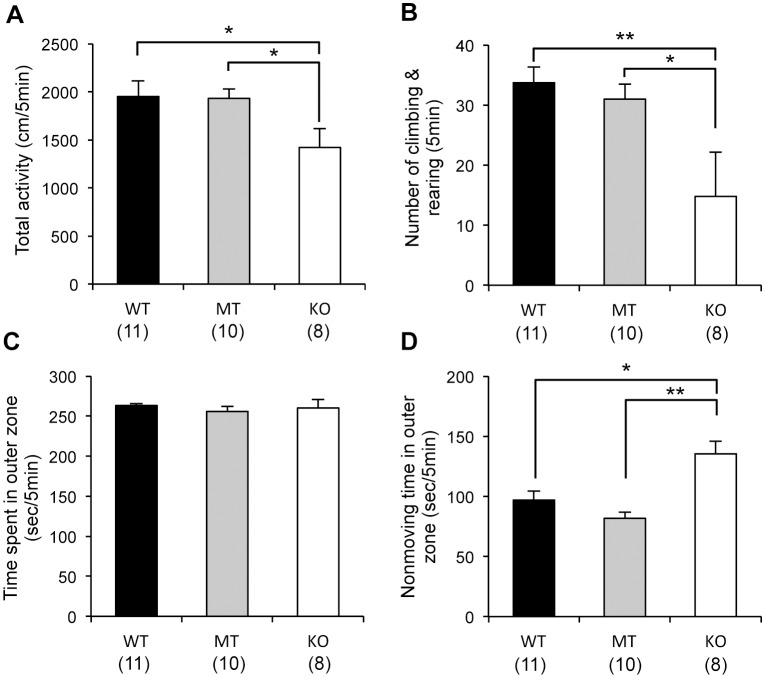
Open field test. Total distance of movement (A), number of climbing and rearing events (B), time spent in the outer zone (C), and resting time in the outer zone (D) of an open field for 5 min. *Usp46* KO mice showed significantly lower values than WT and MT mice for the total distance of movement and the number of climbing and rearing events. Nonmoving time in the outer zone was significantly higher for KO mice. The number of mice used is shown within parentheses. One-way ANOVA with Fisher's PLSD test; *P<0.05, **P<0.01.

The total distance moved and the number of climbing and rearing events was significantly lower for the *Usp46* KO mice than for the WT or MT mice (WT and MT vs. KO, one-way ANOVA, *P*<0.05, F_2,26_ = 3.2 for total activity; WT vs. KO, *P*<0.01; MT vs. KO, *P*<0.05, F_2.26_ = 4.1 for the number of climbing and rearing events) (Fig. 1-A, B). Although no significant differences in the time spent in the outer zone were observed between mice with the 3 genotypes, the KO mice exhibited significantly longer resting times in this zone (WT vs. KO, one-way ANOVA, *P*<0.05; MT vs. KO, *P*<0.01, F_2,26_ = 10.5) (Fig. 1-C, D).

### Ethanol preference test and Ethanol-induced loss of righting reflex test

**Figure 2:**
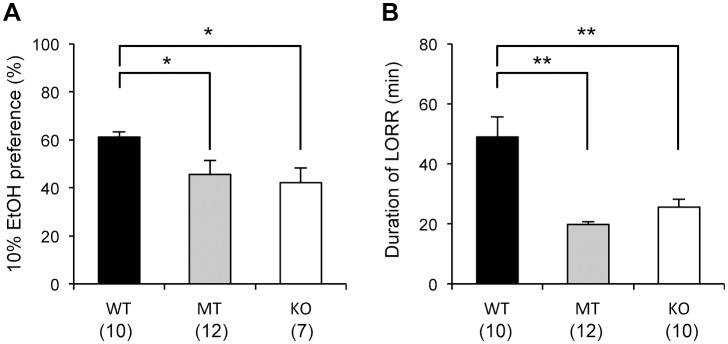
Ethanol preference and ethanol-induced loss of righting reflex test. (A) Ethanol preference (10% ethanol) was significantly lower for *Usp46* MT and KO mice than for WT mice. The ratio was calculated as the volume of ethanol consumed/total volume of fluid consumed per day. (B) Compared to WT mice, both *Usp46* MT and KO mice showed a significantly shorter duration of loss of righting reflex after administration of 3.5 g/kg ethanol (20% v/v in saline intraperitoneally). The number of mice used is shown within parentheses. One-way ANOVA with Fisher's PLSD test; *P<0.05, **P<0.01.

Both the *Usp46* MT and KO mice exhibited lower preference levels for 10% ethanol than the WT mice (WT vs. MT, WT vs. KO, one-way ANOVA, *P*<0.05, F_2,26_ = 4.9) (Fig. 2-A). The mean daily fluid consumption (ethanol+water) per body weight was significantly higher in the KO mice than the MT and WT mice (WT vs. MT or KO, one-way ANOVA, *P*<0.01, F_2,26_ = 9.8) (Fig. S1-A). In the ethanol-induced loss of righting reflex test, the duration of loss of righting reflex was significantly attenuated in both the *Usp46* MT and KO mice compared to WT mice (WT vs. MT or KO, one-way ANOVA, *P*<0.01, F_2,29_ = 6.9) (Fig. 2-B). In both ethanol preference test and ethanol-induced loss of righting reflex test, the scores were not significantly different between *Usp46* MT and KO mice.

### Sucrose preference test

**Figure 3:**
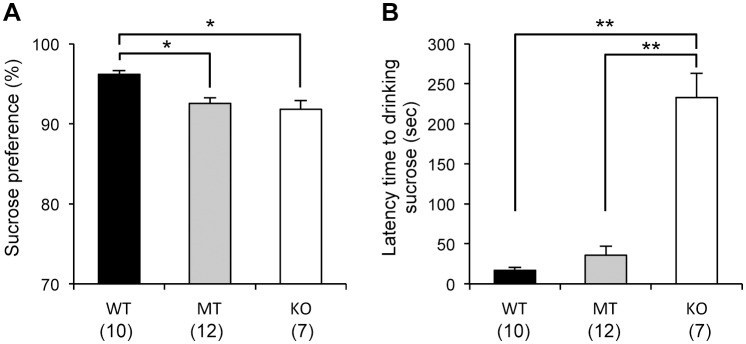
Sucrose preference test and the latency time to drink sucrose. (A) *Usp46* MT and KO mice showed significantly lower preference for 10% sucrose than WT mice did. The ratio was calculated as the volume of sucrose consumed/total volume of fluid consumed per day. (B) On the final day of the sucrose preference test, the latency time to drinking sucrose was measured. *Usp46* KO mice showed a significantly longer latency time than either MT or WT mice did. The number of mice used is shown within parentheses. One-way ANOVA with Fisher's PLSD test; *P<0.05, **P<0.01.

Compared to WT mice, *Usp46* MT and KO mice showed a lower preference for 10% sucrose (WT vs. MT or KO, one-way ANOVA, *P*<0.05, F_2,26_ = 11.5) (Fig. 3-A). The mean daily fluid consumption (sucrose+water) per body weight was significantly lower in the MT mice than the WT mice (WT vs. MT, one-way-ANOVA, *P*<0.01, F_2,26_ = 5.5,) (Fig. S1-B). In this test, we observed that the *Usp46* KO mice did not drink sucrose immediately after the bottle was presented. Therefore, to assess the response to sucrose, we measured the latency to drink sucrose on the final day of the test. The *Usp46* KO mice showed a significantly longer latency to drink sucrose compared to both the *Usp46* MT and WT mice (WT or MT vs. KO, one-way ANOVA, *P*<0.01, F_2,26_ = 66.2) (Fig. 3-B). However, the latency of *Usp46* MT mice was not significantly different from that of the WT mice.

### Novelty-suppressed feeding test

**Figure 4:**
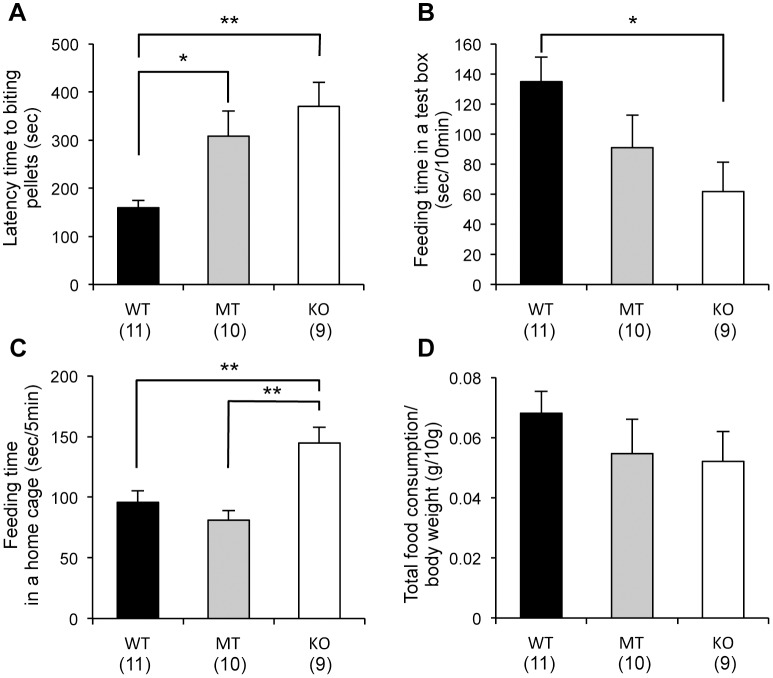
Novelty-suppressed feeding test. (A) *Usp46* MT and KO mice showed an increased latency time to bite pellets in a test box. (B) Feeding time in the test box for 10 min. KO mice exhibited significantly lower feeding times. (C) Feeding time in the home cage for 5 min. The KO mice showed significantly longer feeding times. (D) Total food consumption (in the test box for 10 min, and the home cage for 5 min) was not significantly different between the MT, KO, and WT mice. The number of mice used is shown within parentheses. One-way ANOVA with Fisher's PLSD test; *P<0.05, **P<0.01.

After 24 hours of food deprivation, percentage changes in bodyweight were almost the same in all genotypes of mice (Fig. S2). In the test box, both the *Usp46* MT and KO mice showed a significantly longer latency to bite into pellets (WT vs. MT, *P*<0.05; WT vs. KO, *P*<0.01, one-way ANOVA, F_2,27_ = 9.0) (Fig. 4-A), and compared to WT mice, KO mice showed a significantly lower feeding time during the test period (10 min; WT vs. KO, one-way ANOVA, *P*<0.05, F_2,27_ = 3.6) (Fig. 4-B). However, after returning to their home cage, the KO mice spent significantly longer time feeding than either the WT or MT mice (WT or MT vs. KO, one-way ANOVA, *P*<0.01, F_2,27_ = 12.8) (Fig. 4-C). Total food consumption in the test box and home cage was not significantly different between the 3 mice groups (Fig. 4-D).

### Marble burying test

**Figure 5:**
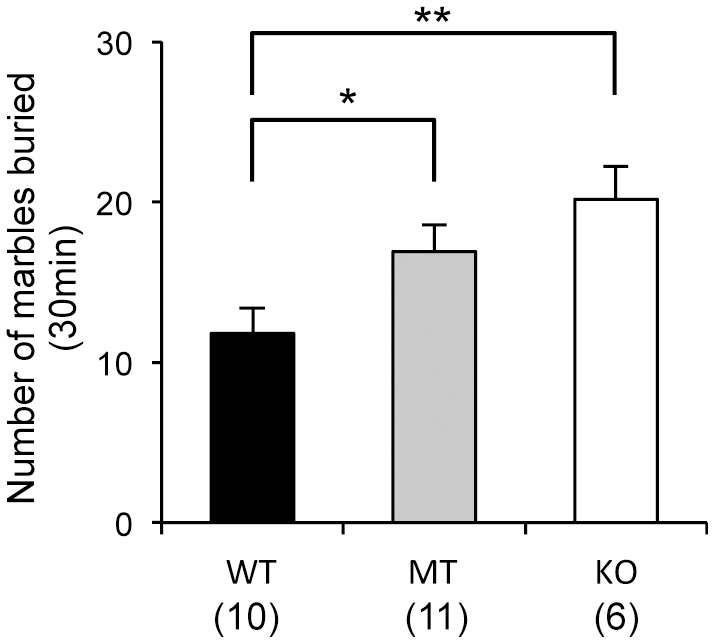
Marble burying test. *Usp46* MT and KO mice buried significantly higher number of marbles than WT mice did. The number of mice used is shown within parentheses. One-way ANOVA with Fisher's PLSD test; *P<0.05, **P<0.01.

Compared to WT mice, both *Usp46* MT and KO mice buried significantly higher number of marbles (WT vs. MT, *P*<0.05; WT vs. KO, *P*<0.01, F_2,24_ = 5.2) (Fig. 5). The differences between *Usp46* MT and KO mice were not significant.

### Novel object recognition test

**Figure 6:**
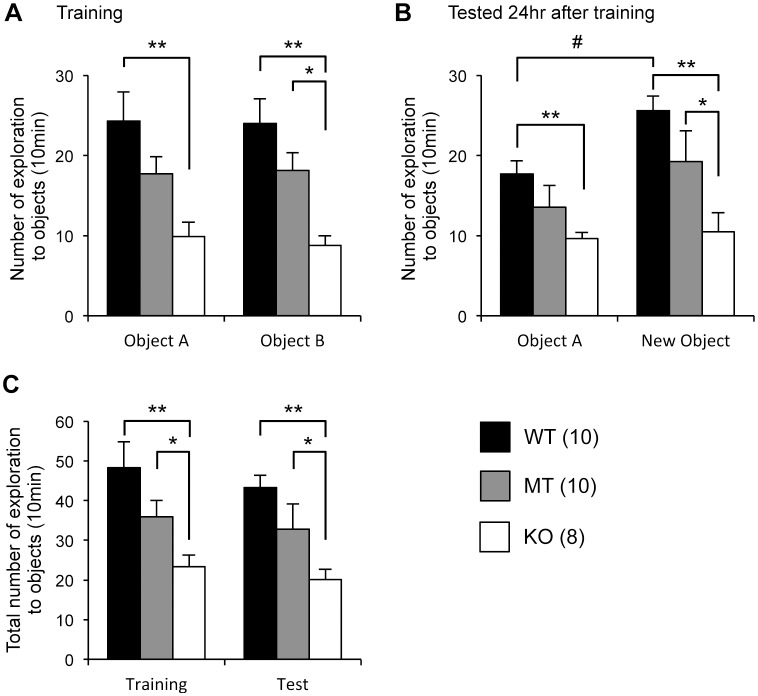
Novel object recognition test. (A) Number of explorations of the 2 objects on the training day. Compared to WT mice, *Usp46* KO mice showed significantly lower numbers of explorations for each object. The number of explorations for object B made by KO mice was also significantly lower than that by MT mice. (B) A familiar object was substituted by a novel object at 24 hours after the training. The number of explorations for the novel object was significantly increased for the WT mice, but not for *Usp46* MT and KO mice. (C) Total number of explorations for the objects on the training day and 24 hours after the training. The number of explorations made by KO mice was significantly lower than those by WT or MT mice. The number of mice used is shown within parentheses. One-way ANOVA with Fisher's PLSD test; *P<0.05, **P<0.01. Student's *t* test; ^#^ *P*<0.05.

On the training day (the third day), no significant differences in the exploration numbers between the 2 objects were observed in any of the genotype groups (Fig. 6-A). However, the number of explorations for each object was significantly lower for *Usp46* KO mice than for WT mice (WT vs. KO, one-way ANOVA, *P*<0.01, F_2,25_ = 7.1 for object A; WT vs. KO, *P*<0.01, F_2,25_ = 11.2 for object B). The MT mice showed an intermediate level between WT and KO mice (MT vs. KO, *P*<0.05 for object B). On the final day (24 hours after training), the WT mice explored the new object significantly more often than a familiar object (student's t-test, *P*<0.05). However, no increase in number of explorations for the new object was observed in either MT or KO mice (Fig. 6-B). The total number of exploration instances for the 2 objects was significantly lower for the KO mice than for the WT mice, and the MT mice showed an intermediate level between the WT and KO mice on both the training and test days (training; WT vs. KO, *P*<0.01, MT vs. KO, *P*<0.05, one-way ANOVA, F_2,25_ = 9.3: test; WT vs. KO, *P*<0.01, MT vs. KO, *P*<0.05, F_2,25_ = 9.6) (Fig. 6-C). When we used time of exploration as a parameter, the same trend as that for numbers of explorations was obtained (Fig. S3).

## Discussion

In our previous study, we reported that TST immobility time for *Usp46* MT mice with a 3-bp deletion coding for lysine is similar to that for *Usp46* KO mice and that the GABA_A_ receptor participates in the regulation of the immobility time [2]. In order to assess the broader functional significance of *Usp46* for behavioral phenotypes that might be related to mental disorders in humans, we subjected *Usp46* MT and KO mice to a variety of behavioral tests. Although behavioral phenotypes of *Usp46* MT and KO mice were not always identical, *Usp46* deficiency significantly affected all behaviors tested. The differences between *Usp46* MT and KO mice were especially distinct with regards to the following parameters: total activity and number of climbing/rearing events, nonmoving time in the outer zone in the open field test, latency time to drinking sucrose in the sucrose preference test, and feeding time in the home cage in the novelty-suppressed feeding test. Because TST immobility time for *Usp46* MT mice was similar to that for *Usp46* KO mice, we thought that the 3-bp mutation of *Usp46* might be a loss of function mutation, not a gain of function mutation in our previous study [2]. However, the present study demonstrated that behavioral phenotypes were not always similar between *Usp46* MT and KO mice, suggesting that this mutation causes less function, not no-function. In fact, it is reported that this mutation does not eliminate total deubiquitinating enzyme activity of USP46 [16].

In the open field test, total activity and number of climbing and rearing events were lower for the *Usp46* KO mice. A lower level of movement was also reflected in the increased nonmoving time in the outer zone. In general, an increased amount of time spent in the peripheral zone of an open field is thought to be an indication of higher anxiety levels [17]. To evaluate the anxiety levels, we measured the time spent in the outer zone. However, mice of all 3 genotypes spent almost the same amount of time in the outer zone, suggesting that the anxiety levels of MT and KO mice are within the normal range. These results are in agreement with those of other behavioral tests that evaluate anxiety levels: no significant differences in the time spent in the open arm of the elevated plus maze among the WT, MT and KO mice (Fig. S4-A, B) and less time spent in the dark compartment of the light-dark box test for the MT mice than the WT mice in our previous study [1].

In the ethanol preference test and ethanol-induced loss of righting reflex test, both *Usp46* MT and KO mice exhibited markedly different phenotypes in comparison to WT mice. Because *Usp46* deficiency results in dysfunction of GABA_A_ receptors [1]–[2] and ethanol is known to act on GABA_A_ receptors [18]–[20], both *Usp46* MT and KO mice are expected to show altered behavioral responses to ethanol. A role for GABA_A_ receptors in influencing ethanol preference has been previously reported in many GABA_A_ receptor subunit KO mice (α1, α2, α5 and δ subunits) [21]–[23]. The altered duration in the ethanol-induced loss of righting reflex test found in our study has also been reported in mice deficient in genes encoding the GABA transporter and GABA_A_ receptor subunits (α1, α2, and β2) [10] [21]–[22]. Thus, our results are consistent with the idea that *Usp46* is involved in the regulation of ethanol-related functions mediated by GABA_A_ receptors.

We performed the sucrose preference test to assess anhedonia, one of the parameters of depression-like responses in mice [24]–[26]. In this test, *Usp46* MT and KO mice displayed a lower sucrose preference, indicating that these mice seem to be behaviorally in a depression-like state. In this test, we noticed that *Usp46* KO mice did not immediately approach a sucrose bottle after the bottle was introduced. In fact, the latency time to drinking the sucrose for KO mice was significantly longer than for the other 2 genotypes. To evaluate the levels of anhedonia and anxiety-related behaviors in more detail, we subjected the mice to the novelty-suppressed feeding test, in which anxiety-induced hyponeophagia, the inhibition of ingestion and approach to food in a novel environment, was examined. In this test, both *Usp46* MT and KO mice displayed longer latency times to start eating pellets, but their total food consumption at the end of the experiment was not significantly different from that of WT mice. These results are consistent with those of the sucrose preference test. Similar phenotypes have been observed in GABA_A_ receptor α2 and γ2 subunit knockout mice [27]–[28]. Although our results may suggest that *Usp46* is involved in the regulation of anhedonic behavior, it is also possible that other mechanisms (e.g. mechanisms regulating metabolism) are implicated in this behavior. With regard to this point, there is room for further investigation because *Usp46* MT and KO mice showed several behavioral phenotypes that do not apply to depression-like behaviors; the immobility time was decreased in both the FST and TST, reflecting mania-like behavior [1], and the anxiety levels were not found to be higher in the open field test and elevated plus-maze test in the present study and in the light-dark box test in our previous study [1]. Thus, the *Usp46*-deficient mouse cannot be characterized as a model mouse for depressive or manic disorders with the present data. Meanwhile, *Usp46*-deficient mice exhibited significantly higher scores than WT mice in the marble burying test. Marble burying behavior is thought to be a pharmacological model for obsessive-compulsive disorder [29], and this behavior is also regulated by the GABAergic system [30]. Taken together, these results suggest that *Usp46* controls a broad range of behavioral phenotypes, and presently, it is difficult to extrapolate the behavioral phenotype of *Usp46-*deficient mice to any specific mental disorders. It also needs further consideration whether different behavioral phenotypes in *Usp46*-deficient mice are due to alterations in GABAergic system. However, our preliminary data that protein expression of several GABA_A_ receptor subunits are altered in several brain regions where *Usp46* is expressed might provide insight into the underlying mechanisms of these behaviors (unpublished data).

Recently, a proteomic study on deubiquitinating enzymes and their associated protein complexes demonstrated that USP46 interacts with PHLPP/SCOP (PH-domain leucine-rich repeat protein phosphatase/suprachiasmatic nucleus circadian oscillatory protein), which are members of the of Ser/Thr family of protein phosphatases [3]. A different study revealed that USP46 functions as a tumor suppressor by binding to PHLPP and directly removes the polyubiquitin chains from PHLPP, resulting in inhibited Akt signaling in colon cancer [31]. Since *Usp46* is intensively expressed in the hippocampus [1], and SCOP has been implicated in the formation of long-term hippocampus-dependent memory [32], we investigated the learning ability of *Usp46*-deficient mice using the same behavioral paradigm as reported previously [32]. In this paradigm, mice are allowed to explore a small field, in which 2 identical objects are placed. Twenty-four hours later, one of the objects is replaced with a novel object. If the mouse remembers the old object, it will spend more time approaching the new one, because mice instinctively interact more with a new object than with a familiar one. Here, we found that *Usp46* MT and KO mice did not explore the new object more than they explored the familiar object. Additionally, these mice spent less time exploring objects than WT mice did on both the training day and the day after training. These results are consistent with a previous report that over-expression of SCOP in the hippocampus blocks long-term memory, but not short-tem memory [32], although we did not examine short-term memory in this study. However, we should note that our results do not necessarily indicate impairment of memory formation. It is possible that the *Usp46*-deficient mice are not able to recognize the object due to some problems with sensory functions, although the retinal histology is normal in these mice (unpublished data). Alternatively, the neophobia-like features of these mice may affect the approaching behavior to the new object. This neophobia-like behavior is observed not only in the novel object recognition test (decreasing approach to the object) but also in the sucrose preference test and novelty-suppressed feeding test (longer latency time to drinking sucrose and to biting pellets).

In summary, the present study has demonstrated that *Usp46* is involved in the regulation of multiple of behavioral phenotypes. Elucidating the function of *Usp46* at both the cellular and central nervous system level may provide further insight into the regulating mechanisms of behavioral phenotypes of *Usp46*-deficient mice which can extrapolate to human mental disorders.

## Supporting Information

Figure S1 **Total fluid consumption in ethanol preference and sucrose preference test.** (A) *Usp46* KO mice showed significantly higher daily fluid consumption (ethanol+water) per body weight (10 g) than WT or MT mice. (B) MT mice showed significantly lower daily fluid consumption (sucrose+water) per body weight (10 g) than WT mice. The number of mice used is given within parentheses. One-way ANOVA with Fisher's PLSD test; **P<0.01.
(TIF)

Figure S2 **Weight changes after 24 hours of food deprivation in the novelty-suppressed feeding test.** Percentage changes of body weight are shown. The number of mice used is given within parentheses.
(TIF)

Figure S3 **Novel object recognition test.** (A) Time of exploration of the 2 objects on the training day. Compared to WT mice, *Usp46* KO mice spent significantly lesser time exploring each object. (B) A familiar object was substituted for a novel object 24 hours after the training day. Time spent exploring the novel object was significantly increased for the WT mice, but not for the *Usp46* MT and KO mice. (C) Total time exploring the objects on the training day and 24 hours after the training. Exploration time for KO mice was significantly lower than that for WT mice. The number of mice used is shown within parentheses. One-way ANOVA with Fisher's PLSD test; **P<0.01. Student's *t* test; ^#^ *P*<0.05.
(TIF)

Figure S4 **Elevated plus-maze test.** (A) Time spent in the open arms during the test period of 6 min. (B) Percentage of time spent in the open arm. The number of mice used is shown within parentheses.
(TIF)
