# Supplementary figures and images for: Behavioral Characteristics of Ubiquitin-Specific Peptidase 46-Deficient Mice
Source: PLoS One. 2013 Mar 5;8(3):e58566. doi: 10.1371/journal.pone.0058566 (PMC3589359; doi:10.1371/journal.pone.0058566)

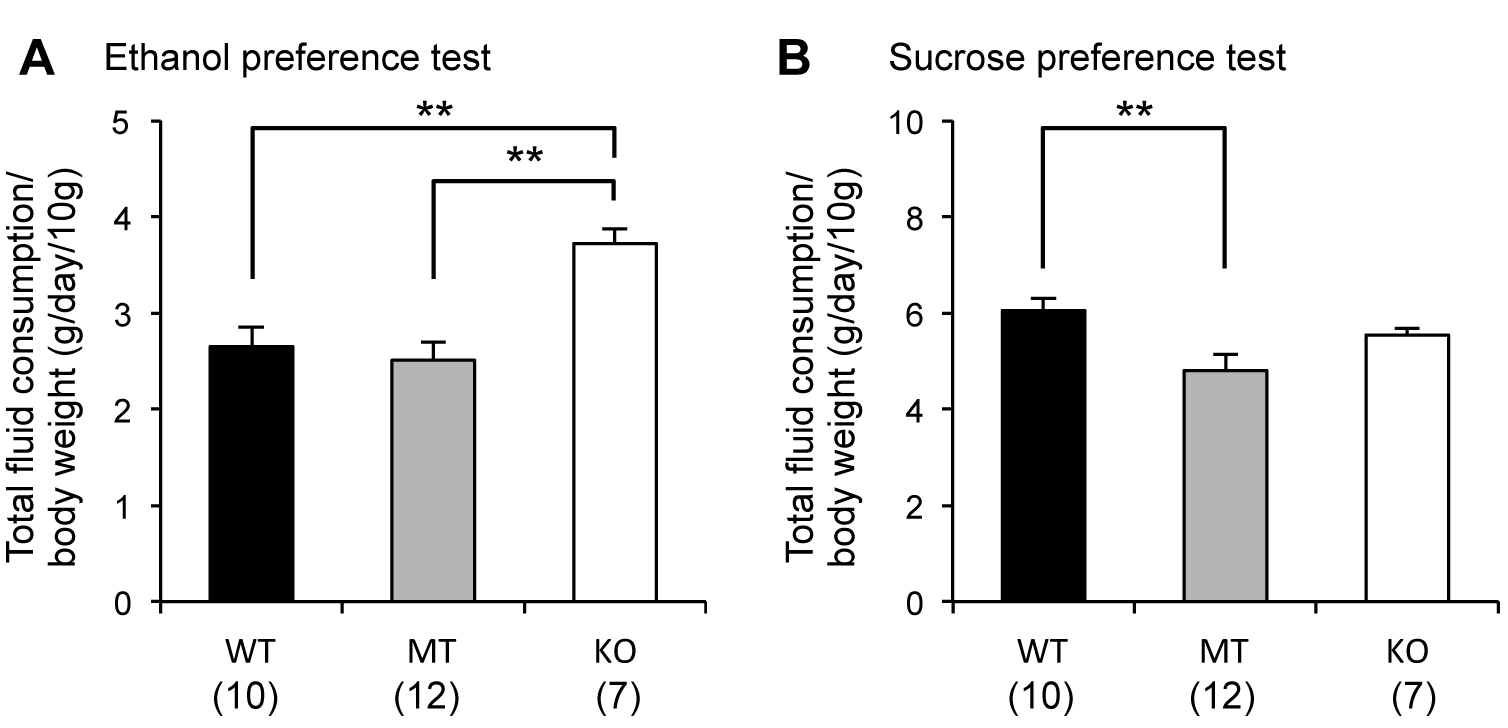

Supplement: Figure S1 — Total fluid consumption in ethanol preference and sucrose preference test. (A) Usp46 KO mice showed significantly higher daily fluid consumption (ethanol+water) per body weight (10 g) than WT or MT mice. (B) MT mice showed significantly lower daily fluid consumption (sucrose+water) per body weight (10 g) than WT mice. The number of mice used is given within parentheses. One-way ANOVA with Fisher's PLSD test; **P<0.01. (TIF) [file pone.0058566.s001.tif]

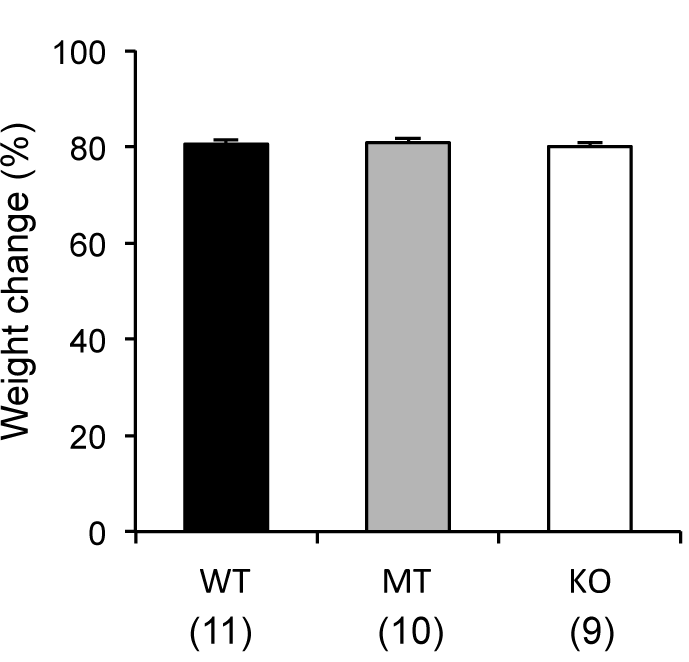

Supplement: Figure S2 — Weight changes after 24 hours of food deprivation in the novelty-suppressed feeding test. Percentage changes of body weight are shown. The number of mice used is given within parentheses. (TIF) [file pone.0058566.s002.tif]

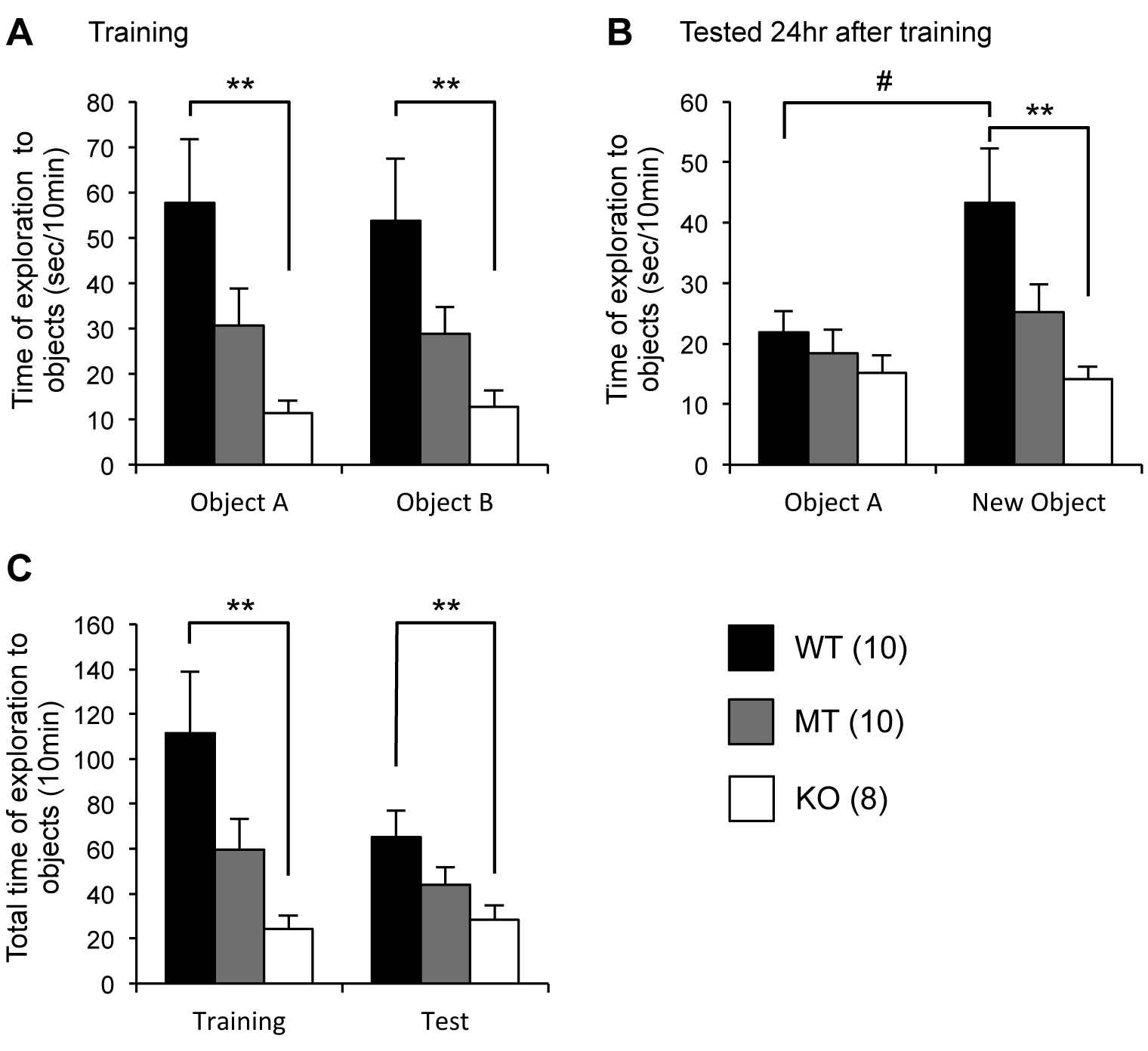

Supplement: Figure S3 — Novel object recognition test. (A) Time of exploration of the 2 objects on the training day. Compared to WT mice, Usp46 KO mice spent significantly lesser time exploring each object. (B) A familiar object was substituted for a novel object 24 hours after the training day. Time spent exploring the novel object was significantly increased for the WT mice, but not for the Usp46 MT and KO mice. (C) Total time exploring the objects on the training day and 24 hours after the training. Exploration time for KO mice was significantly lower than that for WT mice. The number of mice used is shown within parentheses. One-way ANOVA with Fisher's PLSD test; **P<0.01. Student's t test; # P<0.05. (TIF) [file pone.0058566.s003.tif]

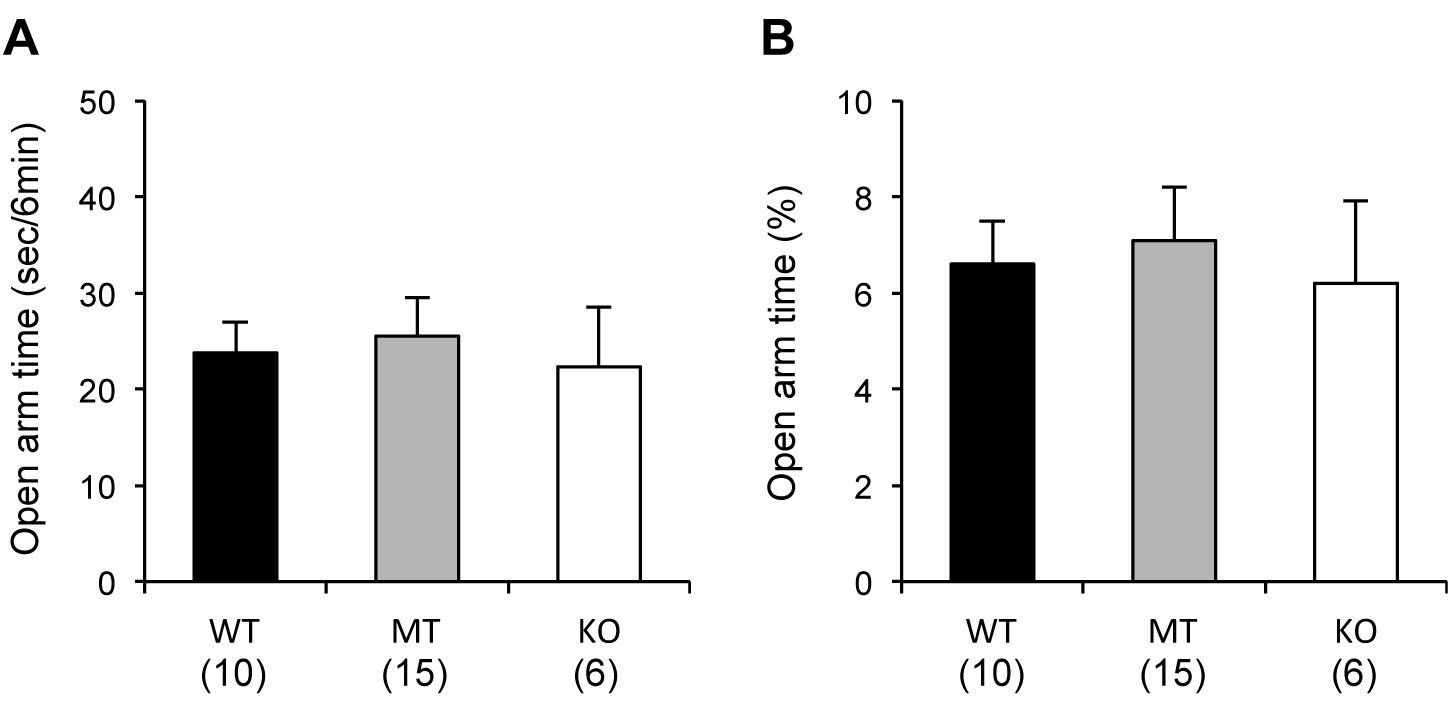

Supplement: Figure S4 — Elevated plus-maze test. (A) Time spent in the open arms during the test period of 6 min. (B) Percentage of time spent in the open arm. The number of mice used is shown within parentheses. (TIF) [file pone.0058566.s004.tif]
